# Lexical Influences on Spoken Spondaic Word Recognition in Hearing-Impaired Patients

**DOI:** 10.3389/fnins.2015.00476

**Published:** 2015-12-23

**Authors:** Annie Moulin, Céline Richard

**Affiliations:** ^1^INSERM, U1028, Lyon Neuroscience Research Center, Brain Dynamics and Cognition TeamLyon, France; ^2^CNRS, UMR5292, Lyon Neuroscience Research Center, Brain Dynamics and Cognition TeamLyon, France; ^3^University of LyonLyon, France; ^4^Otorhinolaryngology Department, Vaudois University Hospital Center and University of LausanneLausanne, Switzerland; ^5^The Laboratory for Investigative Neurophysiology, Department of Radiology and Department of Clinical Neurosciences, Vaudois University Hospital Center and University of LausanneLausanne, Switzerland

**Keywords:** speech perception, lexical influences, word occurrence frequency, hearing loss, aging, spoken word recognition, spondaic words, laterality

## Abstract

Top-down contextual influences play a major part in speech understanding, especially in hearing-impaired patients with deteriorated auditory input. Those influences are most obvious in difficult listening situations, such as listening to sentences in noise but can also be observed at the word level under more favorable conditions, as in one of the most commonly used tasks in audiology, i.e., repeating isolated words in silence. This study aimed to explore the role of top-down contextual influences and their dependence on lexical factors and patient-specific factors using standard clinical linguistic material. Spondaic word perception was tested in 160 hearing-impaired patients aged 23–88 years with a four-frequency average pure-tone threshold ranging from 21 to 88 dB HL. Sixty spondaic words were randomly presented at a level adjusted to correspond to a speech perception score ranging between 40 and 70% of the performance intensity function obtained using monosyllabic words. Phoneme and whole-word recognition scores were used to calculate two context-influence indices (the j factor and the ratio of word scores to phonemic scores) and were correlated with linguistic factors, such as the phonological neighborhood density and several indices of word occurrence frequencies. Contextual influence was greater for spondaic words than in similar studies using monosyllabic words, with an overall j factor of 2.07 (*SD* = 0.5). For both indices, context use decreased with increasing hearing loss once the average hearing loss exceeded 55 dB HL. In right-handed patients, significantly greater context influence was observed for words presented in the right ears than for words presented in the left, especially in patients with many years of education. The correlations between raw word scores (and context influence indices) and word occurrence frequencies showed a significant age-dependent effect, with a stronger correlation between perception scores and word occurrence frequencies when the occurrence frequencies were based on the years corresponding to the patients' youth, showing a “historic” word frequency effect. This effect was still observed for patients with few years of formal education, but recent occurrence frequencies based on current word exposure had a stronger influence for those patients, especially for younger ones.

## Introduction

Speech perception in hearing-impaired patients involves not only the audibility of the speech material but also the entire process of reconstructing meaningful words from partial or deteriorated acoustic input resulting from hearing damage (Miller et al., 1951). This process is dependent on the patient's lexical knowledge (Wingfield et al., 1991; Pichora-Fuller, 2008; Krull et al., 2013), general cognitive ability (Benichov et al., 2012) and on the type of linguistic material used for speech tests (Boothroyd and Nittrouer, 1988; Olsen et al., 1997). Two types of linguistic influence can be distinguished: (1) The type of linguistic material used (e.g., syllables, monosyllabic words, multisyllabic words, or sentences), which has a stronger influence on speech perception as the stimulus becomes more complex (Miller et al., 1951; Boothroyd and Nittrouer, 1988; Olsen et al., 1997) and (2) lexical factors that are well-known to influence speech perception (Goldinger, 1996), such as word occurrence frequency, familiarity, phonological similarity, or age of word acquisition.

Numerous studies have indicated the benefit of contextual information for speech perception in noise (Wingfield et al., 1991; Pichora-Fuller et al., 1995; Pichora-Fuller, 2008). In high-context conditions (high-predictability sentences), contextual information can even compensate almost entirely for moderate hearing loss (Miller et al., 1951; Benichov et al., 2012) and is suggested to be even more beneficial to elderly listeners (Benichov et al., 2012). Most of these studies examined differences between the perception of isolated words and words embedded in sentences with different degrees of predictability, i.e., providing a much higher degree of contextual compensation than isolated words. Sublexical compensation (i.e., compensation at the word level) has also been shown for isolated monosyllabic words, for which older adults can compensate for a loss of word identification in noise by better use of lexical constraints (Boothroyd and Nittrouer, 1988; Nittrouer and Boothroyd, 1990) or in noise-vocoded speech conditions by providing greater exposure to the stimulus to increase word familiarity (Sheldon et al., 2008). Using the contextual influence indices devised by Boothroyd and Nittrouer (1988), the present study explores the main sources of variability in the perception of spondaic words measured in silence, a condition that is much easier for older hearing-impaired patients than the speech-in-noise tasks used in the literature and that is commonly used in audiology to evaluate hearing-impaired patients' speech perception. Speech perception scores can be influenced independently from hearing loss by differences in patients' use of contextual information and by lexical factors (primarily word occurrence frequency and phonological similarity) and the interaction between those lexical factors and patient characteristics.

Indeed, the word frequency effect is one of the strongest and most extensively demonstrated effects in written and, to a lesser degree, spoken word recognition. This effect had been examined using lexical decision tasks (Brysbaert et al., 2011a,b) in young, normal-hearing university students whose characteristics are far from those of the majority of patients usually encountered in audiology clinics, as Benichov et al. (2012) and others have noted. It is likely that in an elderly, hearing-impaired population, linguistic factors have stronger and more heterogeneous effects on word perception scores, especially when accounting for the patients' lexical knowledge and general cognitive abilities. In a task consisting of spoken word repetition in hearing-impaired subjects, the occurrence frequency of the acoustic and phonologic forms of the words, i.e., the spoken word occurrence frequency, is likely to better reflect the subject's relevant spoken word exposure. The greater predictive value of the spoken word occurrence frequency compared with the written occurrence frequency has been noted in written word recognition (Brysbaert and New, 2009). This difference has been attributed to a better match between the type of language material that participants usually read in psycholinguistics experiments and the language of television series and films rather than the more formal language and non-fiction texts represented in the written corpora (Brysbaert et al., 2011a,b). Indeed, the repetition of the spoken form of a word via its frequent availability in real-life situations is likely to aid in its recognition, especially among hearing-impaired older subjects with patchy neurosensory peripheral auditory information. According to this hypothesis, the greatest variability in spoken word recognition would depend on the words' occurrence frequencies at the time of the experiment, independently of the subject's age. However, it could also be argued that older occurrence frequencies might be more relevant to older subjects because age of word acquisition is a predictor of word recognition, albeit a much weaker one than word frequency (Brysbaert et al., 2011a). The spondaic word lists commonly used to assess hearing-impaired patients' speech perception in France date back to the 1950s (Fournier, 1951; HAS, 2007; Legent et al., 2011). Indeed, principles that are still used in speech perception tests in audiology today (for a review: Wilson and McArdle, 2005) were developed in the 1930s (Fletcher and Steinberg, 1929), and Hudgins and Hawkins (1947) developed the first English spondaic word lists in the 1940s. The most important criterion for selecting words, according to Hudgins and Hawkins (1947), was homogeneity with regard to basic audibility, i.e., the words should yield equal perception scores when spoken at a constant level by a normal speaker. Hudgins and Hawkins (1947) suggested that a steeper slope of the performance intensity function reflected greater homogeneity among the words and better precision in graphically obtaining the 50% threshold. The first 42 spondaic word lists were later reduced to the 36 most familiar words by Hirsh et al. (1952). Those principles led Fournier (1951) to select French disyllabic words composed of two equally accented syllables and ending with a vowel sound for his disyllabic words corpus. For greater homogeneity and equivalent difficulty levels among lists, he chose only masculine nouns ending with vowels that were familiar in the spoken day-to-day vocabulary at the time, but he strongly emphasized his regrets about not having a French lexicon database of spoken occurrence frequencies (Fournier, 1951). Thus, because of the natural evolution of language over time, some words that were very frequent in the 1950s are less frequently used today (Michel et al., 2011). This change in language over time provides the opportunity to investigate the hypothesis of a potential historical word frequency effect using several indices of word occurrence frequencies (spoken and written frequencies from different periods (from 1900 to today) on speech perception scores in hearing-impaired patients.

The aim of this study was to explore context influence in spondaic word recognition scores obtained in silence using standard clinical linguistic material in a clinical population. The dependence of contextual influence on characteristics of the linguistic material (mainly word occurrence frequencies) and patient characteristics such as age, ear tested (left vs. right), years of education and hearing loss were examined.

## Materials and methods

### Patients

One hundred sixty patients (75 women and 85 men, aged from 23 to 88 years, mean = 62.1) who were native French speakers and who presented for routine clinical ENT examinations were involved in this study. The patients underwent routine clinical examinations, including otoscopy, tympanometry, pure-tone audiometry at octave intervals from 250 to 8000 Hz and speech audiometry. The patients' number of years of education (YE) ranged from 7 to 17 (mean = 10.6 years) and was obtained from the highest diploma/degree reported by the patients. All of the patients experienced hearing loss after language acquisition, and none presented articulation problems or neurological problems. Most of the patients had noise-induced hearing loss and/or presbycusis (62%), 21% of them presented mixed conductive and sensorineural hearing loss, and 18% presented with sensorineural hearing loss of other origins. Hearing impairments were classified as mild (21–40 dB HL, mean = 31.6, *n* = 72), moderate (41–70 dB HL, mean = 53.0, *n* = 76) or severe (70–90 dB HL, mean = 77.8, *n* = 12), according to the International Bureau for Audiophonology guidelines^1^.

All of the data were anonymously collected, and the study was conducted in compliance with the Helsinki declaration pertaining to human research and the Good Clinical Practice Guidelines. The participants provided written informed consent and the protocol was approved by the French Ethical Committee for Participant Protection (CPP Sud-Est IV).

### Audiological testing

#### Pure-tone audiometry and tympanometry

After a clinical otoscopy examination, the patients underwent pure-tone audiometry using an Interacoustics° AC 40 clinical audiometer in a soundproof booth. Air and bone conduction hearing thresholds, in decibel hearing levels (dB HL), were obtained at octave intervals from 250 to 8000 Hz. For each ear, a four-frequency (500 Hz, 1, 2, and 4 kHz) average pure-tone threshold (PTA) was obtained. Tympanometric measurements were taken using an air pressure from −600 to +300 daPa (Interacoustics° AA222).

#### Spoken spondaic word recognition

Triphonemic monosyllabic word lists currently used in French ENT practices (Lafon, 1964) were presented at several intensities to the patient at minimum steps of 5 dB to obtain the stimulus level corresponding to a phonemic score between 40 and 70% for each patient. Because we wanted to use the exact same presentation level for the monosyllabic words and the disyllabic words, we could not use a stimulus level associated with the 50% threshold; this threshold was quite difficult to obtain using a 5 dB step and presented challenges in terms of time and patients' fatigue. Indeed, because monosyllabic word slopes range from 5%/dB (in normal hearing subjects) to 3%/dB in severely hearing-impaired patients (reviewed for several languages in Han et al., 2009), a minimum of 10–25% variability is expected around the 50% point for a 5 dB variation in stimulus level. Therefore, we chose to accept scores ranging from 40 to 70% (median = 57%, interquartile range at 10%) to obtain a disyllabic whole word score as far as possible from 0 to 100% to avoid floor and ceiling effects.

At this stimulus level, which was kept constant, 60 spondees taken from the Fournier disyllabic word corpus [common clinical material used in France (Collège National d'Audioprothèse, 1999; Legent et al., 2011; Richard et al., 2012) and recommended by the health authorities (HAS, 2007)] were presented in random order to the patients. More than 80% of the variance in the stimulus level was explained by the patients' PTA, thus showing a good adaptation of the stimulus level to the patients' hearing ability. The presentation level for the words averaged 5.6 dB HL (*SD* = 7.6) greater than the patient's average PTA. The pre-recorded words were presented monaurally to the subjects, who were seated in a soundproof booth, and sound levels were monitored using an Interacoustics AC40 audiometer. One ear (left or right) was chosen at random for each patient. The examinees responded verbally after each word presentation, and an experienced audiologist identified the patients' correct and incorrect responses.

### Linguistic analysis of the disyllabic words

The 60 spondees used were extracted from the corpus of 400 spondaic words established by Fournier (1951). The linguistic characteristics of each word were obtained from the Lexique 3.8 database of more than 142,000 words in the French language, which was updated online in October 2012 (http://www.lexique.org) (New et al., 2004, 2007). Most of the words (55/60) were 4 or 5 phonemes long (with an average of 4.5).

#### Occurrence frequency measures

Because not all occurrence frequency estimates are equally predictive (Brysbaert and New, 2009; Ferrand et al., 2010), the occurrence frequency of each spondee has been determined using different metrics, as available in the Lexique° database: the written frequency, based on written texts, and the spoken frequency, based on film subtitles (New et al., 2007). Because we were examining auditory word recognition, we considered the occurrence frequency of a spoken word to be the sum of the occurrence frequencies of each orthographic variant of the same phonological form, and we calculated the cumulative occurrence frequency of all the homophones of each word (for example, most plural forms in French are pronounced the same way as the singular forms; thus, the occurrence frequency of /dragon/ [the same word in English] would be the sum of the occurrence frequencies of /dragon/ and /dragons/). The highest occurrence frequency of all the homophones of each word was also obtained. The frequencies were log transformed, and frequencies lower than 0.01 word per million words (noted as 0.00 in the database) were given a log value of -2.5 (as in Ferrand et al., 2010).

Additionally, we used the word frequencies derived from the Google Books N_gram database (Michel et al., 2011), which provides the frequency of a word's occurrence within published books according to publication year. The word frequencies corresponding to the sum of the frequencies of all of the homophones of each of the 60 spondees were extracted from the Google Books N_gram Viewer, and a smoothing factor of 5 was used to obtain the word frequencies for the years 1900–2005 in 5-year steps.

For each word, the modification rate of the occurrence frequency for that period was calculated. According to this rate, the group of 60 words was split into two groups of 30: the first group (“older words”) had a decreasing occurrence frequency over time (i.e., these words occurred frequently 50 years ago but were much less common now), whereas the second group (“newer words”) comprised words with more stable or increasing occurrence frequencies over time. For each patient, both “older words” and “newer words” scores were obtained.

The word occurrence frequency measures for each word are detailed in the Supplemental Table 1.

#### Phonological similarity measures

To measure the phonological similarities between the stimulus word used and different words in the French language, we used the Lexique° database to calculate the phonological neighborhood of each word, which consisted of the phonological neighbors obtained by substituting a phoneme and the neighbors obtained by deleting or adding a phoneme (Marian et al., 2012). The occurrence frequency of each neighbor was obtained in the same manner that was described above for the stimulus words. Several measurements were calculated using lab-created scripts to characterize each stimulus word: the phonological neighborhood density (the number of phonological neighbors per word) (Luce and Pisoni, 1998), and the high-frequency phonological neighborhood density, defined as the number of phonological neighbors with a higher occurrence frequency than the stimulus word.

### Acoustic analysis of disyllabic words

To rule out a potential confounding factor, i.e., the possibility of an interaction between a word's acoustic spectrum and its linguistic parameters, the spectral acoustic pattern of each spondee was obtained from the recorded versions of the words used, and the root mean square (RMS) amplitude was calculated from 125 Hz to 8 kHz per octave frequency for each of the 60 words used. No statistically significant correlation was obtained between the words' acoustic spectra and the linguistic factors.

### Data analysis

#### Word score measurements

All 160 of the patients tested were included in the analysis. Three of the patients were tested at a level corresponding to a monosyllabic score greater than 70% (88% for one patient). Because the disyllabic word scores of these three patients were considerably less than 100%, we decided to keep them in the analysis. The monosyllabic word scores were used only to determine the stimulation level and were not part of the statistical analysis. For the disyllabic words, the phoneme scores were based on 268 items, the syllable scores on 120 items and the whole-word scores on 60 items.

Because percentage-type variables violated several parametric assumptions (Studebaker, 1985), all of the percentage recognition scores were transformed into rationalized arcsine-transformed scores (or rau scores), which were specifically designed for speech recognition scores (Studebaker, 1985; Sherbecoe and Studebaker, 2004) so that a score of 50 raus corresponds to a percentage score of 50%, and both rau and percentage scores are very close to each other when percentage scores are between 15 and 85%. Although the stimulus intensities were chosen to obtain word percentage scores for each ear that are as close as possible to the middle range (25–75%), thus avoiding floor and ceiling effects, the scores for individual words (calculated across several patients) could exceed 90%. Because most rau units were very close to the percentage scores, only the rau scores are mentioned in the remainder of this manuscript, and percentage scores are only mentioned when they are particularly relevant.

#### Context effect measurements

To evaluate the effects of context on word recognition, we used both the word-to-phoneme score ratios (W/Pho) and the “j factor”, which was defined by Boothroyd and Nittrouer (1988) and is described in Equation (1):

(1) $$\text{Pw}={\text{Pp}}^{\text{j}}$$

with Pw representing the probability of whole-word recognition and Pp representing the probability of recognition of a part of the word (in this case, the phonemes). J varies between 1 (recognition of a single part is sufficient for whole-word recognition) and n (recognition of all the different parts [here, phonemes] is necessary for whole-word recognition). Hence, the j factor can be interpreted as the number of independently perceived constituents of a word, with j approaching 1 as the contextual influence increases.

(2) j=log(phonemic score)/log(word score)

Similar to the method described by Boothroyd and Nittrouer (1988), the j factor was calculated according to Equation 2 only for percent scores between 5 and 95% to avoid extreme values, i.e., for 154 of 160 patients. Because the j factor distribution was not Gaussian, statistical tests were performed on 1/j following a Gaussian distribution using the Shapiro-Wilk test (Shapiro and Wilk, 1965).

To avoid the caveats linked to the tendency for the ratio W/Pho [Equation (3)] to fall as scores approach 100%, both word and phoneme scores were first converted into rau scores:

(3) W/Pho=word scores (in rau)/phonemes scores (in rau)

Because W/Pho did not follow a Gaussian distribution, an arcsine transformation was used to meet the Gaussian distribution requirement for statistical analysis. W/Pho increases (up to 100%) as contextual influence increases: indeed, the more we rely on contextual information, the more we tend to complete patchy sensory information and to increase the number of whole words repeated rather than constituent parts alone (i.e., syllables and phonemes).

#### Statistical analysis

The Gaussian distribution of the data was assessed for each variable using the Shapiro-Wilk test (Shapiro and Wilk, 1965). Pearson's correlations and multiregression analysis were performed for the rau scores obtained for each word across several groups of patients and the linguistic and acoustic characteristics of each word. Correlation coefficients were compared using Fisher's Z-transformed scores (Steiger, 1980). Analysis of variance (ANOVA) and analysis of variance for repeated measures (ANOVA-R) were performed, and the results are presented as the mean and standard deviation (SD). The effect size was measured using Cohen's *d* statistic, η^2^ for ANOVAs (Levine and Hullett, 2002) and correlation coefficients (Cohen, 1992). Following recent statistical guidelines (see for example Asendorpf et al., 2013; Glickman et al., 2014), we used a false discovery rate approach to the problem of multicomparison (Benjamini and Hochberg, 1995; Benjamini and Yekutieli, 2001), with a *p* corrected value of 0.02 (ns for non-significant), to avoid the inflated type II error rate resulting from more classical multicomparison adjustments such as the Bonferroni correction. For the correlational analysis involving hundreds of correlations, random permutation tests (Sherman and Funder, 2009) were used to determine whether the sets of significant correlations observed were due to chance. All of the statistical analyses were performed using R° software, version 2.13.1 (R Development Core Team, 2008), and Statistica° software (StatSoft°).

The analysis involved 2 different approaches. In the first approach, for each word, scores were calculated across the entire population or across different subgroups of patients. In the second approach, scores were calculated for each patient across the 60 words (or across the two groups of 30 words termed “newer words” and “older words”). Several types of patient subgroups were defined within the total population according to hearing loss and/or age and/or years of education (YE) and/or ear tested (right or left) and/or gender. Due to the evolution of education possibilities within the last 80 years in the country, the number of YE showed a decrease as age increased, especially as the same diploma requires more years of education nowadays than 60 years ago. Hence, YE was treated as a dichotomous variable, with a high YE group and a low YE group. There was no interaction between YE group and Ear tested, or PTA, or gender. No statistically significant interactions were detected between Age groups, PTA groups, gender or ear tested (χ^2^ tests are provided in the Supplemental Table 2).

## Results

### Contextual influence on patient scores

As expected, the ANOVA-R and pairwise comparisons between the different scores (monosyllabic word scores and disyllabic word scores calculated in phonemes, syllables and whole words) showed significant differences: *F*_(3, 477)_ > 370, *p* < 0.0001, with the highest scores for disyllabic phonemic scores (mean = 79 rau) and the lowest scores for monosyllabic phonemic scores (mean = 57 rau).

The mean j factor was 2.07 (*SD* = 0.5), and the mean W/Pho = 77.6% (*SD* = 8.9), with a significant correlation between the two (*r* = 0.57, *p* < 0.001). No statistically significant correlations were obtained between j (or W/Pho) and the patients' ages or PTA.

Disyllabic word scores decreased significantly with increasing PTA (*r* = −0.27, *p* < 0.001). However, in our population, no significant relationship was observed between word scores and age, and only a weak relationship was obtained between age and PTA (*r* = 0.23, *p* < 0.005).

**Figure 1:**
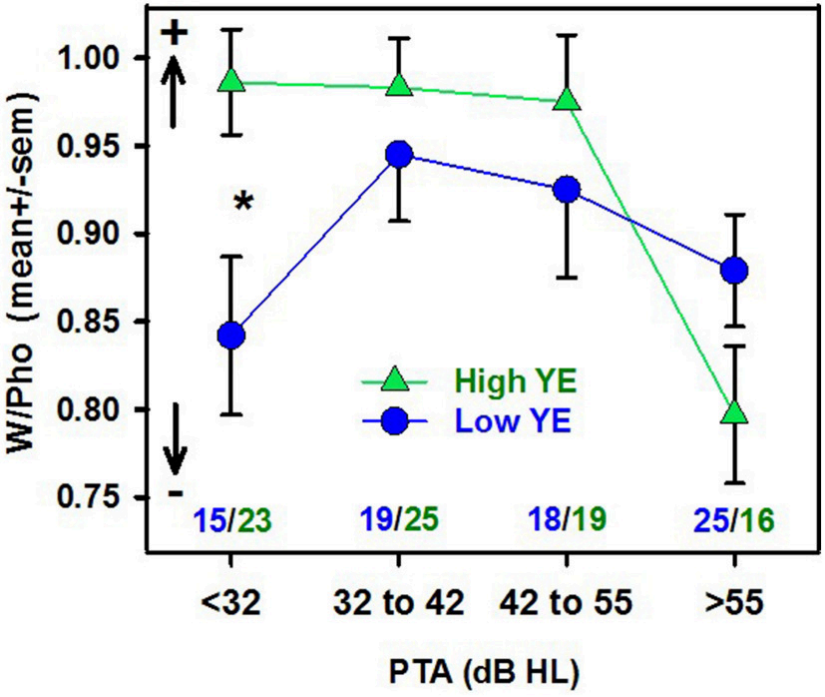
**Mean contextual influence index (W/Pho in arcsine units) as a function of PTA groups (with hearing loss levels specified in dB HL) for the high-YE group (green triangles) and the low-YE group (blue dots)**. The arrows with + and − show how the contextual influence varies. Only significant differences between the YE groups according to the PTA group are shown, with ^*^p < 0.05. The results obtained for the worst-PTA group and the high-YE group differed significantly (*p* < 0.01) from those of the three other PTA groups. The number of patients in each group is shown in blue (low-YE) and in green (high-YE).

When the population was divided into two groups according to YE, W/Pho decreased significantly as PTA increased (*r* = −0.35, *p* < 0.001, *n* = 83) in the high-YE group, with a significant difference compared to the low-YE group (*r* = −0.02, *p* = ns, *n* = 77, *z* = 2.1, *p* < 0.05). For all of the word scores and for W/Pho, there was a significant main effect of PTA and a significant interaction between YE and PTA groups. W/Pho was significantly greater in low-YE patients with mild hearing loss and significantly lower for patients with severe hearing loss (Figure 1). The results of the different ANOVAs are summarized in Table 1. There were no statistically significant influences of YE or PTA on the j factor, although a tendency toward greater contextual influence in the high-YE patient group vs. the low-YE group could be identified [*F*_(1, 146)_ = 2.7, *p* = 0.10].

**Table 1:** **The results of the three ANOVAs performed for the phonemic scores, word scores, and the W/Pho index as a function of the number of years of education (YE, two groups) and the four PTA groups, with Df indicating the number of degrees of freedom, *F* the *F*-values, MSE the mean square error (in gray), *p*-values and the η^2^ measure indicating effect size, and ns indicating non-statistically significant values**.

**Influence of YE and hearing loss on different word scores**										
**Groups**	***Df***	**Phonemic score**			**Word score**			**W/Pho**		
		***F***	***p***	**η^2^**	***F***	***p***	**η2**	***F***	***p***	**η^2^**
YE group	1, 151	0.01	ns	0.00	0.63	ns	0.00	2.02	ns	0.01
PTA group	3, 151	5.54	0.001	0.11	5.89	0.001	0.10	4.73	0.004	0.08
YE X PTA	3, 151	3.48	0.017	0.07	3.61	0.015	0.06	2.98	0.033	0.05
MSE		122.13			180.62			0.03		

**Figure 2:**
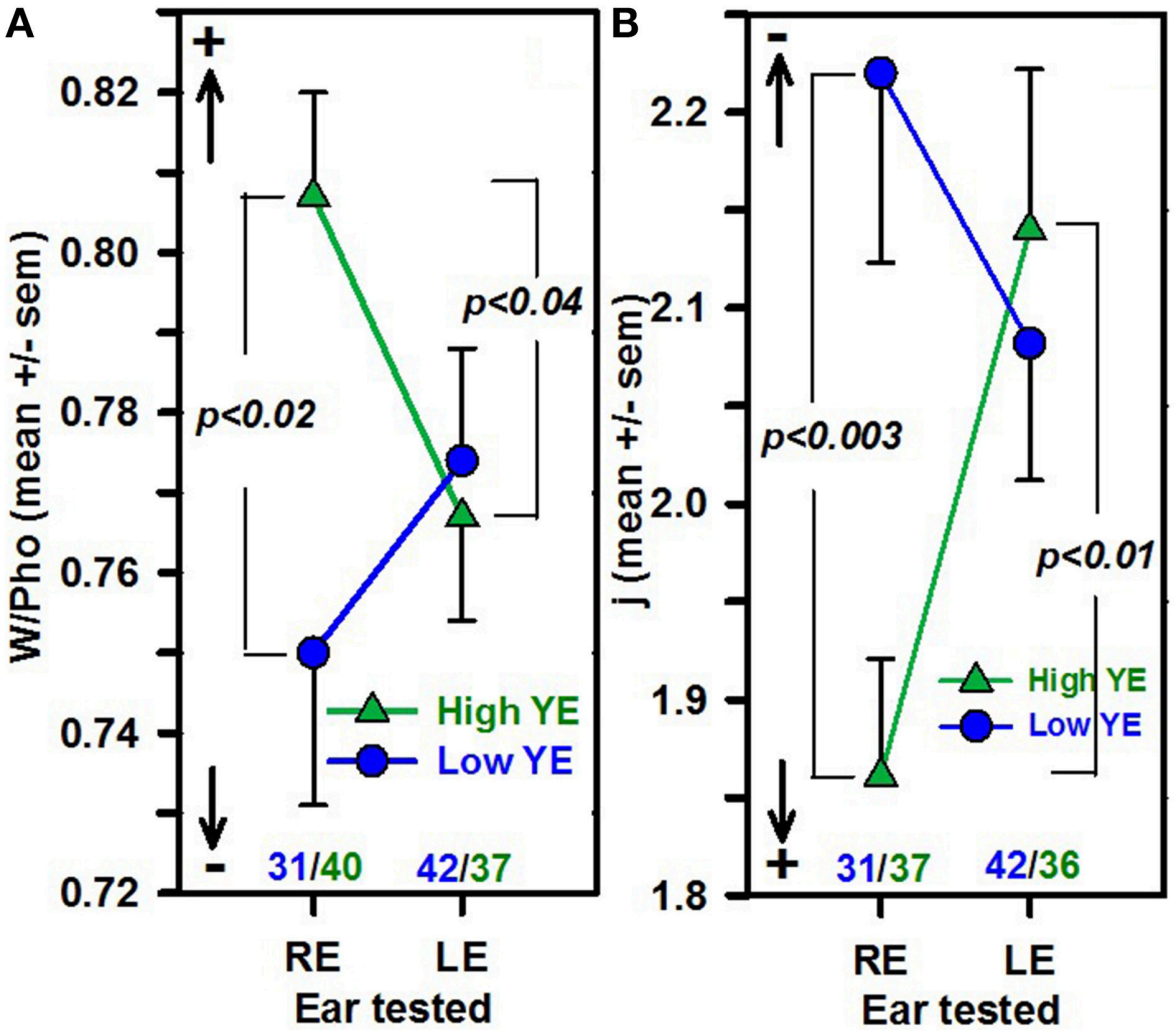
**The mean W/Pho ± SEM (A) and the mean j factor ± SEM (B) as a function of YE (high-YE group in green triangles, low-YE group in blue circles) and ear groups**. The number of patients in each group is noted in blue (low-YE) and in green (high-YE). Arrows with + and − indicate the strength of the contextual influence. Only right-handed patients were selected (*n* = 150).

The influence of the ear tested was analyzed in the subgroup of 150 right-handed subjects and was not statistically significant for any word score [*F*_(3, 146)_ = 0.8, *p* = ns] or for PTA or age. However, there was a statistically significant interaction between YE and the ear tested, with several context indices: *F*_(1, 142)_ = 7.74 (*p* = 0.006, η^2^ = 0.05) for the j factor and *F*_(1, 146)_ = 5.0 (*p* < 0.03, η^2^ = 0.04) for W/Pho, with a significantly greater contextual influence for right ears than for left ears in the high-YE group (Fisher's *t* = 2.7, *p* < 0.007, Cohen's *d* = 0.65 for j) and a greater contextual influence for the high-YE group than for the low-YE group for the right ears (Fisher's *t* = 3.16, *p* < 0.002, Cohen's *d* = 0.78 for j; Figure 2).

When only right ears were selected, a significant difference was obtained between the high-YE and low-YE groups, with the high-YE group having greater contextual influence indices than the low-YE group: one-way ANOVA: *F*_(1, 66)_ = 10.7, *p* = 0.002, η^2^ = 0.14 for the j factor and *F*_(1, 69)_ = 6.8, *p* = 0.01, η^2^ = 0.09 for W/Pho. No significant differences based on the YE or PTA group were obtained for word scores.

### Word linguistic and acoustic characteristics' influences on patients' scores

The percentage score, for each word, calculated across the 160 patients, varied from 17.5 to 92% (word score) and between 49.8 and 95% (phonemic score). Correlations between the word scores and several linguistic factors, such as occurrence frequency (oral and written), phonological neighborhood density, and number of high-frequency phonological neighbors, indicated that occurrence frequency was a major influence and that no other linguistic factors had significant correlations (Table 2). As expected, the correlation between occurrence frequency and word scores was significantly stronger than the correlation between occurrence frequency and phonemic scores (*z* = 2.4, *p* < 0.02), regardless of the occurrence frequency used.

**Table 2:** **Pearson correlation coefficients obtained between word scores, syllabic scores, and phonemic scores (in raus) and word acoustic and linguistic factors**.

**Correlations between word scores and linguistic and acoustic factors**					
	**Variables**	**Whole-word S. (rau)**	**Syllabic S. (rau)**	**Phonemic S. (rau)**	***r* difference, phoneme and word**
Linguistic factors	Lexical spoken occurrence frequency	***0.40***	***0.36***	***0.28***	*p* < 0.01
	Maximum lexical written occurrence frequency	***0.35***	***0.29***	0.21	*p* < 0.001
	Google books occurrence frequency 2005	***0.44***	***0.39***	***0.33***	*p* < 0.001
	Google books occurrence frequency 1950	***0.49***	***0.43***	***0.36***	*p* < 0.001
	Phonological neighborhood density	0.02	0.10	0.11	
	High frequency phonological neighborhood density	−0.18	−0.11	−0.06	
Acoustic factors	0.5 kHz amplitude	***0.37***	***0.42***	***0.53***	*p* < 0.001
	1 kHz amplitude	***0.53***	***0.58***	***0.69***	*p* < 0.001

Significant correlations at the 0.01 level are shown in bold on a gray background, whereas significant correlations at the 0.05 level are shown in bold. Significant differences in correlation coefficients between whole-word scores and phonemic scores are shown in the last column.

The correlations between word scores and occurrence frequency tended to be stronger (but not significantly so) for cumulative oral frequencies than for the maximum occurrence frequency of the phonological form, or the written frequency (*r* = 0.37, *p* < 0.01). Correlations between word scores and occurrence frequencies obtained from the Google Books N_gram French database, calculated in 5-year units from 1900 to 2005, showed the strongest correlations for occurrence frequencies from 1950 (Table 2). However, the differences between the correlation coefficients obtained for the 1950 and 2005 occurrence frequencies did not reach statistical significance.

Significant correlations were obtained between word scores and word amplitude, calculated in RMS per octave, with the strongest correlations for 0.5 and 1 kHz and with significantly stronger correlations for phonemic scores than for word scores (Table 2). No significant correlations were obtained for the frequency band amplitudes centered at 0.25, 2, 4, or 8 kHz. To ascertain the statistical significance and reliability of our correlation results, 50,000 random permutation tests (Sherman and Funder, 2009) were performed on the set of 144 observed correlations to form a distribution of significant findings expected by chance; on average, 9.15 of the 144 observed correlations could have been significant by chance, with an average *r* of 0.10 (*SE* = 0.04). This value is significantly (*p* < 0.0001) below the average *r* observed in the data (0.34) and lower than the number of significant correlations observed (60), showing that the pattern of correlations observed here cannot be attributed to chance.

Stepwise regression analysis starting with five potential explanatory variables representing acoustic factors (0.5 and 1 kHz amplitude in dB) and linguistic factors (occurrence frequency and phonological neighborhood density) yielded statistically significant models that could explain 40% of the variance of word scores and 54% of the variance of phonemic scores (Table 3) using only two explanatory variables, 1 kHz amplitude and occurrence frequencies. Occurrence frequency had a greater influence on word scores (beta = 0.58), and 1 kHz amplitude had a greater influence on phonemic scores than occurrence frequency did (beta = 0.68 vs. 0.25).

**Table 3:** **Stepwise multiregression analysis of the word scores (top table) and phonemic scores (bottom table) as a function of several explanatory variables, representing acoustic factors (0.5- and 1-kHz amplitudes) and linguistic factors (lexical spoken occurrence frequency and phonological neighborhood density)**.

**Word Score (rau)**	***R*^2^**	***B***	***SE***	**Beta**	***F***	***t***	***P***
**REGRESSION ANALYSIS OF WORD AND PHONEMIC SCORES**							
Step 1	**0.28**				23		< 0.0001
Intercept		−7.7	14.77			−0.5	ns
1 kHz amplitude		1.6	0.33	0.53		4.8	< 0.0001
Step 2	**0.4**				21		< 0.0001
Intercept		−11.7	13.4			−0.9	ns
**1 kHz amplitude**		1.5	0.29	**0.38**		3.8	< 0.0005
**Lexical spoken Oc. Freq**.		8	2.12	**0.52**		5.1	< 0.0001
**PHONEMIC SCORE (RAU)**							
Step 1	**0.48**				53		< 0.0001
Intercept		10.1	9.54			1.1	ns
1 kHz amplitude		1.54	0.21	0.69		7.3	< 0.0001
Step 2	**0.54**				34		< 0.0001
Intercept		8	9			0.9	ns
**1 kHz amplitude**		1.5	0.2	**0.68**		7.6	< 0.001
**Lexical spoken Oc. Freq**.		4.1	1.4	**0.25**		2.8	< 0.01

Only the significant predictors are noted, and only those models that are significantly different from the previous one are presented (variance analysis between models with p < 0.01). The model coefficients are specified as B (with standard error in SE) and standardized coefficients are noted as Beta (in bold). Each model is specified by the percentage of variance explained (r^2^, in bold) and the corresponding degree of statistical significance (p), with ns for non-significant. The degree of significance of each predictor at each step is noted with t and p.

### Influence of the interactions between patients and word characteristics on speech perception scores

**Figure 3:**
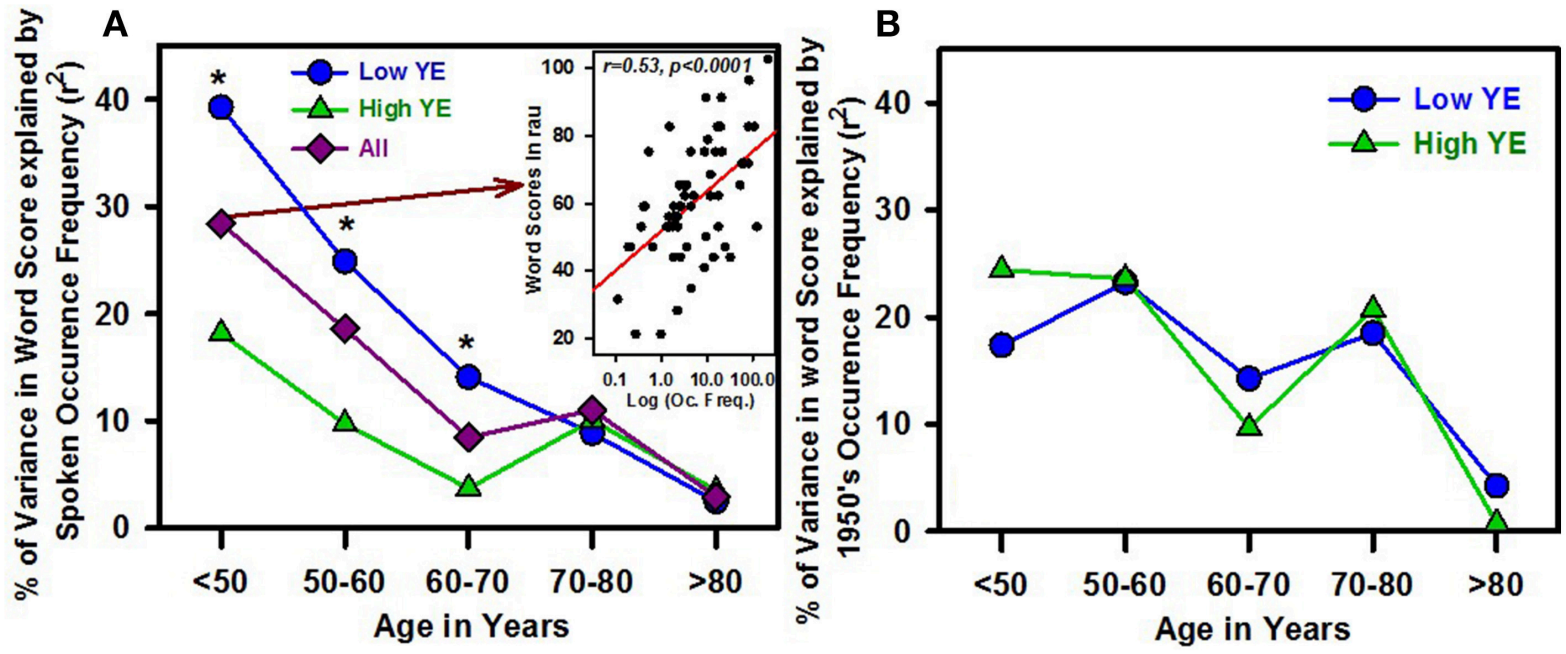
**Percentage of variance in word scores explained by the spoken language occurrence frequency calculated from the Lexique° database, as a function of the patients' age (five different groups), for the subgroup of patients with a low number of educational years (low YE, blue dots), the patients with a high number of educational years (high YE, green triangles) and the total group of patients (all, dark red diamonds)**. The stars indicate statistically significant differences in the percentage of variance explained between the high- and low-YE groups (difference in correlation coefficients measured by *z*-scores). An example of correlation between word scores in rau (obtained for all patients under 50 years old) and occurrence frequency (in log units) is shown in the inset figure in **(A)**. **(B)** shows the percentage of variance in word scores explained by the 1950s language occurrence frequency taken from the N_gram Google Books database.

The percentage score for each word was calculated for different groups of patients organized by age and/or number of years of education. A strong effect of age was observed, with significantly stronger correlations between word scores and word frequencies for the youngest group (under 50 years of age; *r* = 0.53, *p* < 0.0005) than for all groups older than 60 years of age (*r* = 0.33, *p* < 0.02; *z* = 2.6, *p* < 0.01; Figure 3A). The same analysis, performed by grouping the patients by age and YE, revealed a significantly stronger relationship between word scores and word frequencies for the low-YE group than for the high-YE group (with significant differences for all three groups under 70 years of age). The low-YE group exhibited systematically higher correlation coefficients than the high-YE group. A similar result was obtained for W/Pho, which showed a decreasing correlation as age increased, especially for the low-YE group. The effects of age on the dependency of patient responses to current spoken language occurrence frequencies might be related to the fact that those words were relatively more common in the 1950s than today.

**Figure 4:**
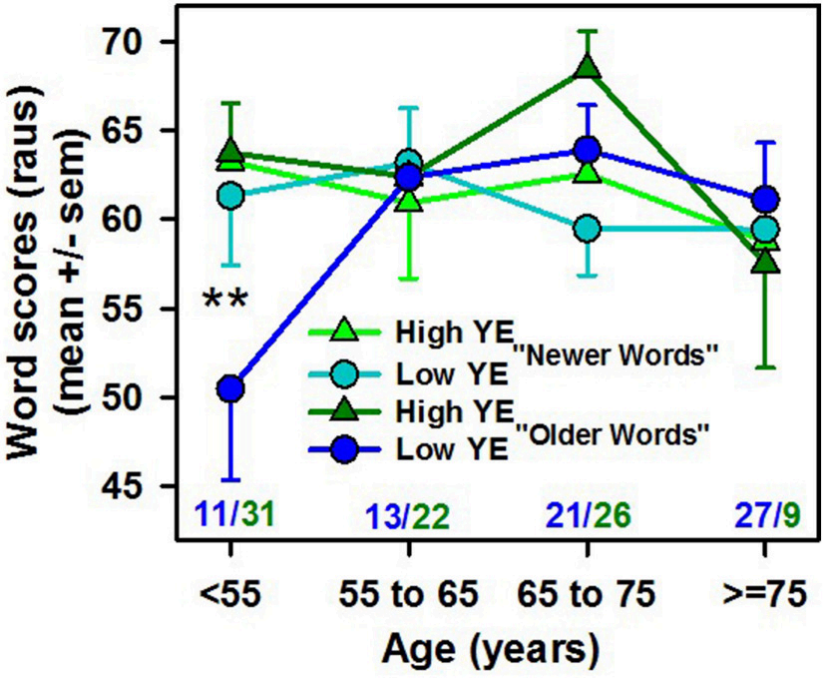
**Word scores in raus (mean ± SEM) for the subgroup of “older words” (words whose occurrence frequency decreased over time) and “newer words” (words whose occurrence frequency remained stable or increased over time) calculated for each patient as a function of the patient's age in years (specified in the x-axis) and number of years of education (high YE in green and low YE in blue)**. ^**^p < 0.01 in the *post-hoc* analysis. The number of patients in each group is noted in blue (low-YE) and green (high-YE). The interaction between age and word group was statistically significant [*F*_(3, 152)_ = 4.3, *p* < 0.006].

To check this hypothesis, we calculated “older words” and “newer words” scores for each patient (Figure 4). A mixed-ANOVA (1 within-subjects factor: word group, and 2 between-subjects factors: YE and age groups) showed no significant difference according to each variable (YE, age or word group), but there was a significant interaction between word group and age: *F*_(3, 152)_ = 4.3, *p* < 0.006, ${}_{p}\eta^{2}=0.08$. The interaction between word group, age and YE was not statistically significant [*F*_(3, 152)_ = 2.4, *p* = 0.07]. For the youngest and low-YE patients, the “older words” scores were significantly lower than (1) the scores of the older patients and high-YE patients and (2) the “newer words” scores (Figure 4). In addition, only the low-YE patient group showed a statistically significant correlation between age and the difference between the “older words” and “newer words” scores (*r* = −0.39, *p* < 0.0005 for the low-YE group and *r* = −0.07, *p* = ns for the high-YE group), with a decreasing difference as age increased that was mostly related to an increase in the “older words” score as age increased. This could explain why the younger patients, especially those with low *YE* values, were more sensitive to the current spoken word occurrence frequencies from the Lexique° database. Both the age and YE effects disappeared when the 1950s occurrence frequencies were used (Figure 3B).

**Figure 5:**
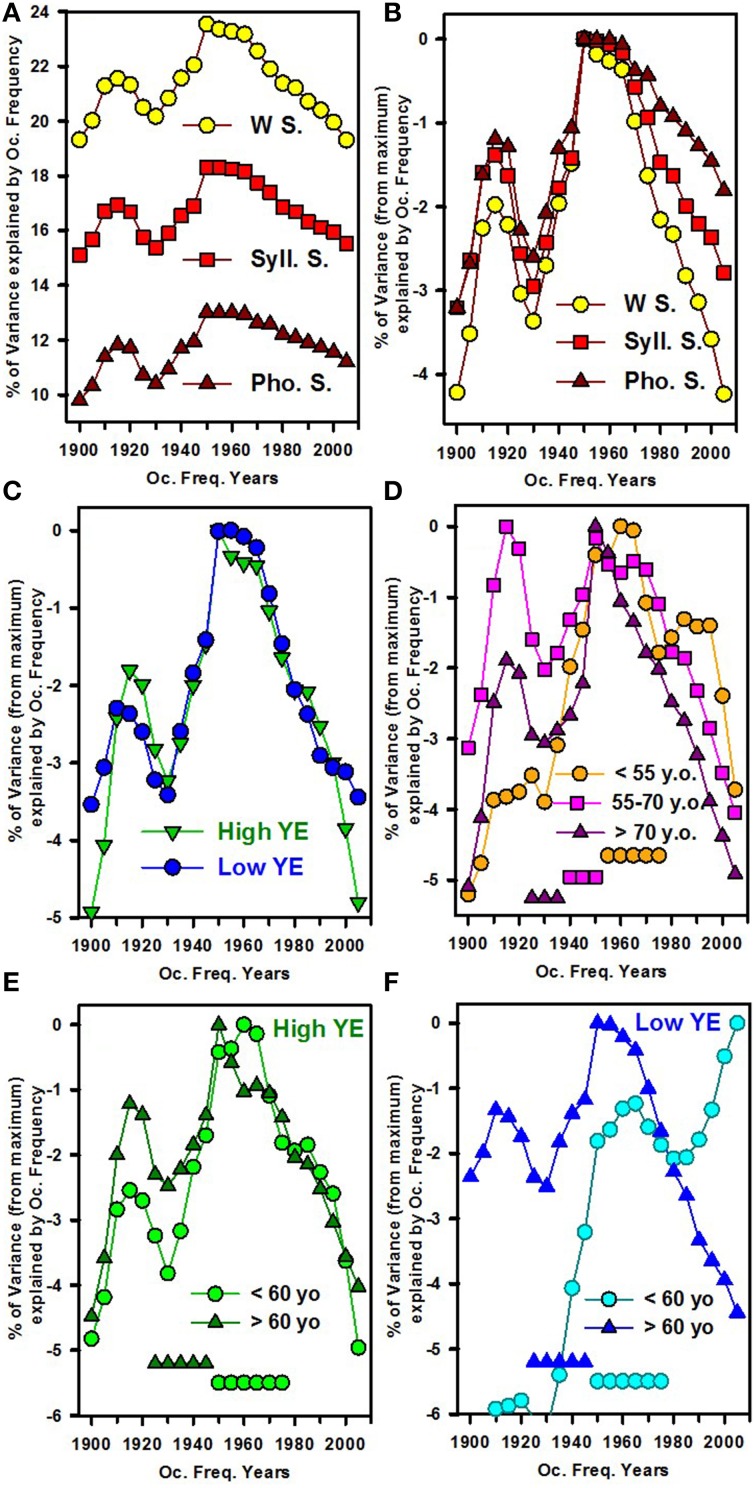
**Percentage of variance in the word scores (y-axis) explained by word occurrence frequencies measured in different years (in 5-year bins, from 1900 to 2005, x-axis)**. For **(B–F)**, the maximum percentage of variance was normalized to zero so that the other values show the decreases in the percentage of variance explained by word occurrence frequencies during periods other than the optimal period. **(A)** Percentage of variance in the whole-word scores (W scores, yellow dots), the syllabic scores (Syll. scores, red squares) and the phonemic scores (Phonemic scores, dark red triangles), explained by word occurrence frequencies per 5 years. **(B)** Shows the same data; however, the maximum percentage of variance was normalized to zero, allowing the comparison of the different patterns of variance as a function of year. **(C)** Normalized percentage of variance in the whole-word scores for the group of patients with a high number of years of formal education (high YE, green triangles) and those with a low number of years of formal education (low YE, blue dots). **(D)** The normalized percentage of variance in the word scores of different age groups, with dark purple triangles for the eldest patients, pink squares for those 55–72 years old and orange dots for the youngest patients (under 55 years of age). The patients' birth dates are represented by the symbols on horizontal lines parallel to the x-axis. **(E,F)** The normalized percentage of variance in the whole-word scores for the two different age groups, with dark triangles for the oldest patients (over 60 years of age) and light dots for the youngest patients (under 60 years of age). The birth dates are represented by symbols on horizontal lines parallel to the x-axis. **(E)** Shows the subgroup of patients with the highest number of years of education (high YE, in green), whereas **(F)** depicts the subgroup of patients with the lowest number of years of education (low YE, in blue).

To investigate which occurrence frequency explains the greatest variability in the word scores, correlations among word scores, phonemic scores and the occurrence frequencies obtained from the N-gram database in 5-year units were analyzed. For the entire group of patients, the best correlations were observed for the occurrence frequencies from 1950 to 1960: Figure 5A depicts the percentages in variance in word, syllabic and phonemic scores, explained by occurrence frequency as a function of the year in which the books were published. To compare evolution as a function of the year, the maximal percentage of variance across the years was set at zero so that the other percentages showed the amount of decrease in the percentage of variance explained by the different occurrence frequencies (Figure 5B). Hence, Figure 5B shows that the word scores appeared more dependent on the occurrence frequencies years than syllabic or phonemic scores did. The decrease in the dependence of scores on occurrence frequency for recent years showed a greater slope for word scores than for phonemic scores. When the total population was split by YE, the pattern was very similar (Figure 5C). However, a clear age effect occurred when we grouped the patients by age: younger patients were more sensitive to more recent occurrence frequencies, whereas older patients were more sensitive to “older” occurrence frequencies (Figure 5D). An interaction between YE and age was again observed, with a greater difference between the young and old patients in the low-YE group (Figure 5F) than in the high-YE group (Figure 5E).

## Discussion

### Contextual influence measures of disyllabic words

The phoneme scores for disyllabic words were 17 raus greater on average than the word scores, which allowed the calculation of different contextual influence indices. Both the j factor and the average ratio of word scores to phonemic scores (W/Pho) across our population showed greater contextual influence than the data reported by Olsen et al. (1997) (with *j*-values ranging from 2.3 to 2.8) and by Boothroyd and Nittrouer (1988) for young, normally hearing university students using monosyllabic consonant-vowel-consonant (CVC) words presented in noise with a 0-dB SNR (78 vs. 72.3% for W/Pho and 2.07 vs. 2.46 for j factor). Because the j factor varies between 1 (word perceived as a whole) and the maximum number of parts used as measurement units (in this case, phonemes), its range depends on the type of linguistic material used. Whereas both Olsen et al. (1997) and Boothroyd and Nittrouer (1988) reported j factors that ranged between 1 and 3 (because of their use of CVC words), our j factors could theoretically have ranged between 1 and 4.5 (the average number of phonemes in our disyllabic words). Therefore, a j factor of 2.07 denoted a substantially greater contextual influence on the word scores, which was confirmed by our higher W/Pho. Although contextual influence tends to increase with YE, the greater contextual influence in our population compared with the reports in the literature cannot be attributed to a higher YE because our population was heterogeneous regarding YE and had an average YE much lower than that of the subjects in most studies. The most likely explanation is the greater redundancy of 4- to 5-phoneme disyllabic words than triphonemic monosyllabic words. Additionally, because the j factor can be interpreted as the number of chunks of information independently perceived by the listener, our 2.07 j factor can speculatively be interpreted as agreeing with the disyllabic structure of the words used: the patients tended to perceive the words as two individual syllables rather than as a string of phonemes, in agreement with the greater syllabic structure of the French language compared with English (Ferrand et al., 1996, 2010).

### Differences in contextual influence depending on patients characteristics

#### Years of education

The contextual influence on spondee recognition was significantly greater in the high-YE than in low-YE patients, especially those with milder hearing loss (< 32 dB HL), which underlines the importance of YE to spoken word recognition scores in an elderly, hearing-impaired population in an audiological clinic. YE can be considered a very crude reflection of lexical knowledge and cognitive ability. Indeed, in a meta-analysis, Verhaeghen (2003) reported a significant correlation between YE and two vocabulary tests (the WAIS-R vocabulary subtest and the Shipley scale). Although contextual influence tended to be greater in the high-YE patient group, major differences between both groups appeared in combination with hearing loss. The high-YE group with an average PTA lower than 55 dB HL was better able to repeat complete words (with significantly greater contextual influence, as shown by their higher ratio of word scores to phonemic scores) than the low-YE patients. This finding suggests that these patients exhibited better compensation for the partial phonological information they receive using top-down lexical information, at least in cases of mild to moderate hearing loss. Such compensation was not observed for patients with severe hearing loss (PTA > 55 dB HL). A likely explanation resides in the overly degraded auditory information available to patients with more severe sensorineural hearing loss, who experience greater distortions, widened cochlear filters, frequency selectivity alteration and loss of temporal resolution (Moore, 2007) that cannot be compensated for by a simple increase in the absolute stimulus level. This heavily degraded auditory information would not be sufficient to properly fuel the lexical restoration process. This finding is consistent with the results of Başkent et al. (2010), who demonstrated that perceptual phonemic restoration could be identified in normal-hearing subjects and those with mild hearing impairments, but not in patients with moderate hearing loss (PTA > 40 dB HL in patients over 60 years of age in their study). Similarly, Benichov et al. (2012) observed a decrease in contextual benefit for their patients with moderate hearing loss (PTA > 45 dB HL) compared with patients with mild hearing loss. The results of the present study indicate that the degree of compensation for the degraded bottom-up information, using top-down lexical processes, varied greatly from patient to patient, even for the recognition of isolated words in silence, with a heavy emphasis on general vocabulary knowledge reflected by years of formal education.

#### An age effect?

The lack of an age effect on contextual influence indices that was observed in this study appears to contrast with the results of Krull et al. (2013), who observed that the top-down restoration process declined with age in an identification task involving isolated monosyllabic words in speech-shaped noise. However, this finding could be attributed to several factors: the population described in our study consisted of a majority of older patients (50% of the patients were over 65), so that our younger patients were actually substantially older (i.e., the 10th percentile of our population was 42 years old) than the young group of Krull et al. (which was between 18 and 32 years of age). Second, the task used in our study consisted of the auditory recognition of disyllabic words in silence and used words with different degrees of linguistic difficulty, whereas Krull et al. (2013) used monosyllabic words presented in noise and showed an age-related decrease in the ability to exploit temporal and spectral glimpses embedded in words presented in speech-shaped noise. The use of disyllabic words, which offers greater information redundancy than monosyllabic words, is likely to have favored a “lexical restoration” process in our high-YE patients without specific noise-induced perception difficulty that would have negated the benefits of the restoration process. Saija et al. (2014) showed that although normal-hearing older participants (average age: 66 years) exhibited poorer speech intelligibility in interrupted noise than a younger group (average age: 22 years), the older patients maintained phonemic restoration even better than the young group. The authors hypothesized that the process of speech perception degradation in noise with age could be counteracted by top-down processes dependent on the increased general knowledge and vocabulary observed in elderly subjects compared with younger participants (Park et al., 2002; Keuleers et al., 2015). Additionally, using full sentences as stimuli, Saija et al. (2014) provided their participants a broad range of mostly linguistic cues, including syntactic and semantic contexts in addition to lexical cues, that could be used for speech restoration. This better use of contextual information by older adults has been observed in several studies (Wingfield et al., 1991), especially when adding semantic clues (Boothroyd and Nittrouer, 1988; Nittrouer and Boothroyd, 1990; Sommers and Danielson, 1999; Pichora-Fuller, 2008). This is consistent with the hypothesis of compensation for the decrease in fluid intelligence with age, by maintenance of, or improved use of/an increase in general knowledge, including linguistic and verbal knowledge, as encompassed by the crystallized intelligence concept (Cattel, 1963; Horn and Cattell, 1966). More recently, Rogers et al. (2012) presented a more pessimistic view: they showed that the greater use of contextual information by older adults is more likely to lead to false hearing in incongruent semantic conditions than among younger subjects. Hence, the greater benefit (or compensation) from contextual information would be related to the older adults' tendency to respond in a manner consistent with the context and not necessarily to better use of contextual information because the former leads to more errors when the context is misleading.

#### Left/right ear

The present results suggest that the degree of compensation from sublexical influence was not only educational level-specific, hearing loss-dependent and, to some degree, age-dependent (in terms of occurrence frequency), it is also ear-dependent and has a significant interaction with YE; there was a significantly stronger contextual influence for words presented in the right ears than in left ears in the high-YE patient group among the subgroup of 150 right-handers, with no significant differences in hearing loss, age or word scores between the right and left ears. Moreover, when only the right ears were considered, the high-YE group exhibited significantly greater contextual influence than the low-YE group on the two context influence indices. The so-called right ear advantage linked to hemispheric functional asymmetry for language processing is usually observed behaviorally when both ears are competing, i.e., words presented in a subject's right ear are more likely to be repeated than words presented concomitantly in the left ear at a comfortable loudness level (for a review, see Lazard et al., 2012). Here, the situation was very different: the task was monaural, and its difficulty stemmed from the low sound level used, which was adjusted to obtain a word score of approximately 50% in a hearing-impaired population. The absence of an ear difference in the raw word scores with the presence of a right-ear advantage for contextual influence, argues in favor of the involvement of higher-level processing and not a peripheral effect. Among the many studies examining speech perception in noise and speech restoration, very few have specifically investigated the difference between right and left ears. Pisoni et al. (1970) obtained more efficient recall of sentences with semantic constraints presented in a noise masker in the right ears vs. the left ears of right-handed subjects, suggesting a right-ear advantage in contextual influence that is linked to cerebral dominance. In speech perception evaluations, hearing-impaired patients are tasked with building a meaningful auditory word from patchy phonological information, i.e., a task that is very close to phonemic restoration (Warren, 1970) and to the Ganong effect (Ganong, 1980), in which ambiguous speech sounds are properly categorized when presented in a word context, showing evidence of reciprocal interaction between phonetic and lexical processing. The neural correlates of phonological-lexical interactions have been preferentially shown in the left hemisphere with the involvement of the left supramarginal gyrus and left middle temporal gyrus (Prabhakaran et al., 2006; Myers and Blumstein, 2008). Using the Ganong effect, Gow et al. (2008) reported an increase in phonetic activation in the left posterior superior temporal gyrus within the time frame associated with a lexical effect, providing evidence in favor of a top-down feedback model and allowing for a direct influence of the lexical context on phonemic perception rather than only a post-perceptual decision process. Using prior knowledge of the speech content to enhance the clarity of degraded speech, Sohoglu et al. (2012, 2014) argued further in favor of an early influence of linguistic knowledge on the top-down modulation of acoustic processing. The greater contextual effect observed in our patients' right ears vs. left ears could be attributed to the left hemispheric preference for phonological-lexical interaction processing, with a preference for left hemisphere-right ear top-down interaction. However, ear preferences for the monaural presentation of auditory stimuli would be best investigated in an intra-subject paradigm, which would imply the use of word stimuli carefully balanced between both ears.

### The word frequency effect viewed through the looking-glass of the speech perception scores of hearing-impaired elderly patients

Because the words used in the present study had a broad range of occurrence frequencies and included some older words whose frequency of use has greatly diminished over the years, the influence of the patients' age on the relationship between word occurrence frequency and word score seemed particularly relevant. Indeed, we observed that the dependence of spoken word recognition on the spoken word occurrence frequency decreased as age increased. This finding appears to contradict several results showing a greater dependence on word frequency in spoken word recognition as age increases (Revill and Spieler, 2012), which is consistent with most studies of visual word recognition. In those studies, a stronger predictive value of written word frequency has been observed in older subjects than in younger subjects (matched for vocabulary size and YE) (Spieler and Balota, 2000; Balota et al., 2004). However, the population observed here differed in several major respects from populations in other studies: our younger subject group was far older than the typical young subjects in those studies (university students), and our population had an average educational level that was lower than that of the university graduates who are usually included as study participants. Indeed, when the data were analyzed according to YE group, the dependency on word occurrence frequency was significantly greater for the low-YE group than for the high-YE group, and the age effect, i.e., the greater occurrence frequency dependence for younger groups, disappeared for the high-YE group. This outcome could be attributed to an increased learning advantage and the greater and longer exposure to words experienced by older adults than younger adults; the older adults had a larger vocabulary and greater familiarity with words that were frequent in the 1950s but that are rare today. The statistically significant differences between the low-YE and high-YE groups among the younger subjects reinforced this hypothesis, with word frequency showing significantly greater predictive power in low-YE groups, who had lower scores for rare words.

In addition, not all occurrence frequency estimates are equally predictive (Brysbaert and New, 2009). Our results showed that the spoken word frequencies, which were obtained from a film subtitle database, explained 16% of the variance vs. 12.2% for the written book frequencies, confirming the superiority of the film subtitle database over written frequencies. However, word occurrence frequency is not the only parameter that influences word recognition (Goldinger, 1996), and its influence is difficult to separate from those of age of acquisition and word familiarity (or subjective frequency). Because most of our patients were over 50 years of age, and the word lists used here came from a corpus designed in the 1950s (Fournier, 1951), a number of the words that appeared to be unfamiliar to younger subjects (in their twenties to forties) were more familiar to an elderly population because the elderly people had encountered those words in their younger years. Thus, perhaps historical word occurrence frequencies, dating back to the youths of these elderly patients, could better explain their scores than current word occurrence frequencies. The potential influence of the “occurrence frequency year” was suggested by Brysbaert and New (2009); however, Brysbaert et al. (2011b) reported no decrease in predictability among older subjects in a lexical decision task with the use of the most recent occurrence frequencies, which were taken from the Google Books N_gram database (Michel et al., 2011).

The discrepancy between our results and the lack of an influence of the occurrence frequency year reported by Brysbaert et al. (2011b) can be explained by at least two factors: (a) the populations studied were very different: the data reported by Brysbaert et al. (2011a,b) involved two groups of patients, including older adults, both with high YE (data from Spieler and Balota, 2000), whereas the present study revealed a “historical occurrence frequency” effect that was more important for the low-YE group, and (b) the present study examined auditory word recognition in hearing-impaired patients, vs. visual word recognition with a lexical decision task which was used in Brysbaert et al. (2011b). Auditory word recognition may be more sensitive to the historical word frequency effect than visual word recognition. Indeed, by reanalyzing correlations between Luce and Pisoni (1998) auditory perceptual data and the more recent occurrence frequency databases, Yap and Brysbaert^2^ showed that auditory word recognition tended to be more sensitive to the age of acquisition than visual word recognition was. Thus, the “historical occurrence frequency effect” observed in the present study might be attributable in part to the stronger effect of age of acquisition on auditory word recognition than on visual word recognition. We observed that the 1950s occurrence frequencies tended to be better predictors of the word scores than the spoken occurrence frequencies obtained from the Lexique 3.8° database (24 vs. 16% of variance explained), and they were better predictors than the more recent N-gram frequencies (2005), which explained 19% of variance. Additionally, the 1950s occurrence frequencies explained a significantly greater percentage of the variance for both contextual influence indices that we used: W/Pho (28 vs. 22%) and j. When the 1950s occurrence frequencies were used, the age effect on the relationship between word scores and occurrence frequency disappeared. When we correlated word scores with the historical occurrence frequencies from 1900 to 2005, the greatest variance explained was obtained for the 1950s; this variance had a similar shape regardless of whether the group was divided into high- or low-YE groups. However, when the scores were grouped by the patients' ages, the peak of the explained variance shifted toward more recent years (1970) for the younger patients. This effect was observed for both the low-YE and high-YE groups. For the low-YE patients under 60 years of age, the maximal percentage of variance appeared for the most recent occurrence frequencies (2005); for the older group, the maximal percentage of variance occurred for the occurrence frequencies from the 1950s.

This result suggests that exposure to a word at a younger age seems to have greater impact than current exposure does, perhaps because of a stronger and more stable mental representation. Additionally, because most of our patients suffered from presbycusis with gradually worsening hearing loss over their lifetime, it is possible that exposure to a word's phonological form at a younger age was more relevant because it corresponds to an exposure to a less-degraded stimulus, i.e., exposure occurred at a time when the hearing loss was milder or even non-existent, thus contributing to building a stronger mental representation. This historical word frequency effect may be emphasized in hearing-impaired patients compared with non-hearing impaired subjects, which would explain why it was not observed systematically in Brysbaert et al. (2011b).

### Potential implications for audiology practice

This study extends the main results of psycholinguistic research concerning the influence of linguistic context on spoken word recognition to the speech scores obtained from a heterogeneous hearing-impaired population similar to the population encountered in clinical practice, with potential consequences for speech scores. Indeed, the task most commonly used to evaluate speech perception in audiology, i.e., the repetition of a heard word with no time constraints, differs from the tasks usually used in word recognition research (i.e., reaction times/scores in lexical decision and naming tasks), and the population tested here (i.e., a hearing-impaired, older population with great variability in linguistic and general knowledge) differs from the typical university student cohorts used in psycholinguistics studies. Indeed, even for isolated words presented in silence, contextual influences can add substantial variability to speech scores. Top-down lexical compensation (or the lack thereof) for partial phonological information can greatly increase inter-subject variability depending on the patient's YE, age, hearing loss and ear tested. The influence of the ear tested was only visible for contextual influence indices and not for raw scores; thus, it is probably negligible in practice compared with other factors. The historical word occurrence frequency effect, which was of variable importance depending on the patients' age and number of years of education, suggests a strong interaction between linguistic factors and patient-specific factors. This interaction emphasizes the need to consider linguistic factors carefully (including the “history” of these factors) when developing speech recognition material (and to avoid focusing only on acoustic factors) (Meyer and Pisoni, 1999). Although achieving perfect item equivalence in speech perception linguistic material across several variables for a heterogeneous patient population could be considered wishful thinking, the current availability of large lexical databases encompassing several languages and types of occurrence frequencies is allowing substantial improvements in the current material used.

## Conclusion

Substantial inter-subject variability related to contextual influences can be identified in the speech perception scores for spondaic words in audiological clinic populations. These influences vary according to patient-specific factors, such as hearing loss characteristics, age, ear tested (right/left ear), and years of formal education. These patient-specific factors interact differently with linguistic material-specific factors, such as the occurrence frequency and phonological similarities of words. This phenomenon is illustrated by the historical occurrence frequency effect observed here, in which spondaic word recognition scores showed a stronger correlation with the word occurrence frequencies corresponding to the patient's youth than with current word occurrence frequencies; the older hearing-impaired patients were more likely to repeat a word that is rarely heard now but was common in their youth than a word that occurs frequently in daily communications (i.e., a word to which they are strongly exposed) but was rare in their youth. This finding was especially true for patients with more years of education. Even at the isolated word level, when words are presented in silence, lexical influence can partially compensate for bottom-up loss of phonological information in mild to moderate hearing loss and can improve spondaic recognition scores, but it depends strongly on general and linguistic knowledge.

### Conflict of interest statement

The authors declare that the research was conducted in the absence of any commercial or financial relationships that could be construed as a potential conflict of interest.

## Footnotes

^1^International bureau for audiophonology Audiometric Classification of Hearing for Impairments. In BIAP Recommendation n° 02/1 bis. Available online at: http://www.biap.org.

^2^Yap, M. J., and Brysbaert, M. (2009). *Auditory word Recognition of Monosyllabic Words: Assessing the Weights of Different Factors in Lexical Decision Performance*. Available online at: http://crr.ugent.be/papers/Yap_Brysbaert_auditory_lexical_decision_regression_final.pdf.
